# Adsorptive–Photocatalytic Composites of α-Ferrous Oxalate Supported on Activated Carbon for the Removal of Phenol under Visible Irradiation

**DOI:** 10.3390/molecules29153690

**Published:** 2024-08-04

**Authors:** Salomé Galeas, Víctor H. Guerrero, Patricia I. Pontón, Carla S. Valdivieso-Ramírez, Paul Vargas-Jentzsch, Paola Zárate, Vincent Goetz

**Affiliations:** 1 Doctoral School Energy and Environment, University of Perpignan Via Domitia (UPVD), 52 Avenue Paul Alduy, 66100 Perpignan, France; salome.galeas@epn.edu.ec; 2 PROcesses Materials and Solar Energy, PROMES-CNRS UPR 8521, Rambla de la Thermodynamique, 66100 Perpignan, France; 3 Department of Materials, Escuela Politécnica Nacional, Ladrón de Guevara E11-253, Quito 170525, Ecuador; victor.guerrero@epn.edu.ec (V.H.G.); patricia.ponton@epn.edu.ec (P.I.P.); carla.valdivieso@epn.edu.ec (C.S.V.-R.); 4 Department of Nuclear Sciences, Escuela Politécnica Nacional, Ladrón de Guevara E11-253, Quito 170525, Ecuador; paul.vargas@epn.edu.ec (P.V.-J.); paola.zarate@epn.edu.ec (P.Z.)

**Keywords:** organic pollutants, ferrotitaniferous sands, adsorption–photocatalysis synergy, metal–organic framework, visible-light assisted photocatalysis

## Abstract

Adsorptive–photocatalytic composites based on activated carbon (AC) and α-ferrous oxalate dihydrate (α-FOD) were synthesized by an original two-step method and subsequently used for the removal of phenol from aqueous solutions. To obtain the composites, ferrotitaniferous black mineral sands (0.6FeTiO_3_·0.4Fe_2_O_3_) were first dissolved in an oxalic acid solution at ambient pressure, and further treated under hydrothermal conditions to precipitate α-FOD on the AC surface. The ratio of oxalic acid to the mineral sand precursor was tuned to obtain composites with 8.3 and 42.7 wt.% of α-FOD on the AC surface. These materials were characterized by X-ray powder diffraction, scanning electron microscopy, and the nitrogen adsorption–desorption method. The phenol removal efficiency of the composites was determined during 24 h of adsorption under dark conditions, followed by 24 h of adsorption–photocatalysis under visible light irradiation. AC/α-FOD composites with 8.3 and 42.7 wt.% of α-FOD adsorbed 60% and 51% of phenol in 24 h and reached a 90% and 96% removal efficiency after 12 h of irradiation, respectively. Given its higher photocatalytic response, the 42.7 wt.% α-FOD composite was also tested during successive cycles of adsorption and adsorption–photocatalysis. This composite exhibited a reasonable level of cyclability (~99% removal after four alternated dark/irradiated cycles of 24 h and ~68% removal after three simultaneous adsorption–photocatalysis cycles of 24 h). The promising performance of the as-prepared composites opens several opportunities for their application in the effective removal of organic micropollutants from water.

## 1. Introduction

In recent decades, several technologies have been proposed to deal with the challenges derived from water pollution. Frequently, a single remediation approach cannot alone solve the problem of water contamination, especially when dealing with persistent and recalcitrant compounds. As a result, the idea of using a combination of processes has gained special attention, and numerous efforts have been made in this regard [1,2,3,4]. For instance, merging adsorption and heterogeneous photocatalysis has demonstrated a synergy in the removal of water pollutants, including emerging contaminants [5,6], antibiotics [7,8,9,10,11], and dyes [12,13,14,15], using for this a wide variety of materials such as metal–organic frameworks [16], Z-scheme composites [17], and coordination polymers [18].

Among the advantages of using adsorptive–photocatalytic composites is the dual functionality that allows them to address the intermittence of solar light; thus, during the night, pollutants can be adsorbed on the surface of the composite, while heterogeneous photodegradation can occur under the sunlight [10]. Furthermore, the recovery of the photocatalyst powder is easier when it is supported onto a bulk adsorbent [19,20].

Porous carbon-based materials, including biochar and activated carbon, are considered conventional adsorbents that are highly effective and convenient for the removal of a wide variety of organic compounds due to their high surface area, porosity, ease of recovery, relatively low cost, and surface functionalization [10,21,22]. Furthermore, recent studies have demonstrated a synergistic effect when combining these carbonaceous materials with photocatalysts, enabling the pollutants to be adsorbed near the photocatalytic active sites, and maximizing the photodegradation process [23,24]. 

In an effort to benefit from solar energy as an innocuous, free, and highly available light source, recent studies have incorporated visible light-active photocatalysts supported on porous carbon-based materials [5,25,26]. This is due to the possibility of exploiting the majority of the visible light component (~43%) rather than the UV fraction (<5%) of the solar spectrum [27]. For example, several authors have produced activated carbon composites with titanium dioxide doped with compounds like Er^3+^:YFeO_3_ [28], tungsten [29], fluorine and nitrogen [26,30,31], and bismuth [32]. These doping agents have provided a visible-light active character to the TiO_2_; however, they are expensive and, in some cases, not innocuous. 

α-ferrous oxalate dihydrate (α-FOD) is a metal–organic framework (MOF) that has been cataloged as a novel visible light photocatalyst [33,34,35]. According to our previous studies [36,37], we have demonstrated the feasibility of synthesizing α-FOD from the reaction of a low-cost mineral precursor (Ecuadorian ferrotitaniferous sands, 0.6FeTiO_3_·0.4Fe_2_O_3_ [38]), working with an environmentally friendly method that uses oxalic acid under subcritical water conditions as the reaction medium. In addition, it was found that depending on the synthesis conditions such as temperature, oxalic acid concentration, amount of ferrotitaniferous sand, and reaction time, α-FOD together with titanium dioxide (TiO_2_) were obtained (i.e., T = 135 °C, 1M oxalic acid, 1.75 g of precursor, and 4 h) [36]. This suggests that tailoring the synthesis conditions opens the possibility of obtaining pure α-FOD.

Also, the photocatalytic activity of α-FOD synthesized from black mineral Ecuadorian sands has been demonstrated. As such, α-FOD with an estimated bandgap of 2.31 eV (536 nm) was able to photodegrade phenol from water under visible light irradiation, achieving a removal efficiency of ~93% within 6 h [37]. Therefore, in this work, a novel approach is reported for the first time for the synthesis of AC/α-FOD composites with dual adsorptive–photocatalytic functionality. AC obtained from coconut husk as a natural waste source was used effectively as a support for the visible light-responsive photocatalyst α-FOD. Ferrotitaniferous black sands were employed to synthesize AC/α-FOD composites with two different concentrations of the photocatalyst compositions by varying the amount of mineral sand precursor. The as-synthesized composites were characterized by scanning electron microscopy (SEM), X-ray powder diffraction (XRPD), and the nitrogen adsorption–desorption method. Adsorption and photocatalytic tests under visible light were performed to highlight the two functionalities of the composites. Also, repeatability of adsorption–photocatalysis tests were conducted during several cycles to evaluate the partial or total regeneration of the adsorbent, which is a decisive property to assure a suitable response of the composite for real applications in removing organic pollutants.

## 2. Results and Discussion

### 2.1. Synthesis and Characterization of AC/α-FOD Composites

#### 2.1.1. Synthesis

AC/α-FOD composites were synthesized by the reaction of iron–titanium mineral sands (precursor) with an oxalic acid solution and the subsequent precipitation of the photocatalyst onto activated carbon, as detailed in Section 3. By adjusting the load of the precursor (1 g or 6 g), composites containing 8.3 ± 2.0% and 42.7 ± 2.1% of α-FOD were obtained and named CS1g and CS6g, respectively (see the end of Section 3.3, where the calculation of these percentages is described in detail). It is worth mentioning that a concentration of 1.5 M of oxalic acid led to the formation of pure α-FOD, avoiding the precipitation of TiO_2_ on the composites. This is of significant importance because the ultimate purpose of these adsorptive–photocatalyst composites is the possibility of using them in solar light applications, as mentioned before. 

#### 2.1.2. Crystalline Structure

**Figure 1:**
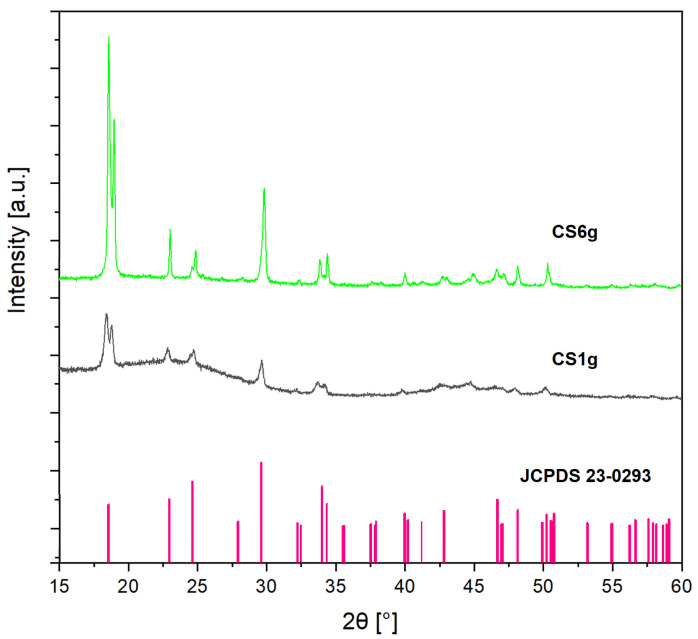
XRPD patterns of the as-synthesized composites (lines of α-FOD correspond to those of JCPDS card 23-0293).

The XRPD patterns of the as-synthesized composites are displayed in Figure 1. These results show that both composites exhibit peaks corresponding to monoclinic ferrous oxalate dihydrate (α-FOD), similar to JCPDS card 23-0293 [39]. Usually, AC is an amorphous material that shows broad peaks around 2θ = 24° and 43° [12,40]. As the AC percentage in CS1g composite is approximately 91.7%, the characteristic peaks of amorphous AC are observed in the baseline of the XRPD pattern, whereas the lower content of AC in CS6g (approx. 57.2%) confirms that the predominant phase observed is the α-FOD.

#### 2.1.3. Morphology and Surface Characteristics

**Figure 2:**
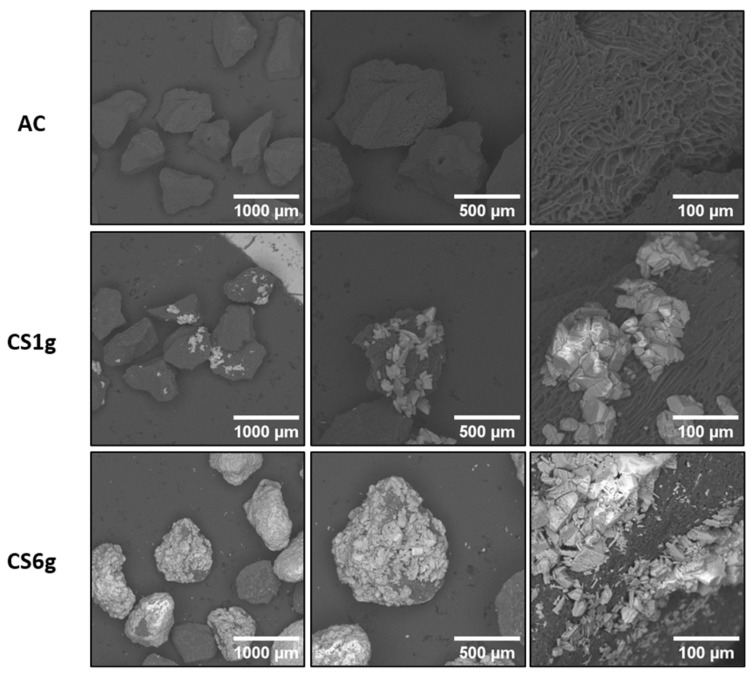
SEM images of the AC and the AC/α-FOD composites.

SEM images of the AC and the as-synthesized composites at different magnifications are shown in Figure 2, where the brighter structures correspond to α-FOD and the dark zones to the AC. The images reveal the irregular morphology and porous structure of the AC, as well as the heterogeneous distribution of the photocatalyst onto the AC surface for both composites. Agglomeration of the α-FOD particles is evident in the two composites and has been reported by other authors for similar composites [41,42]. The surface of CS6g was highly coated by α-FOD compared to that of CS1g, as expected from the high load of precursor used in the synthesis. It is important to note that partial coating of the AC surface is desirable for the expected double functionality of the composites. While the pollutant can keep easy access to sites of adsorption within the meso/microporous network on the AC, photodegradation of the contaminant can take place by the action of the α-FOD. The presence of AC did not significantly affect the morphology of α-FOD particles during the synthesis; in both composites, the predominant shape was bi-pyramidal, as reported previously in other works [37,43,44]. Interestingly, for CS6g, besides the bipyramidal shape, some relatively smaller prismatic structures were evident. This could be associated with the difference in iron concentration in the solution during the first step of the synthesis. CS6g, for instance, was obtained with a high precursor load, which led to a higher iron concentration in the reaction medium. Thus, for CS1g, the nucleation may have taken less time to complete than for CS6g, which could have influenced particle growth [41]. Conversely, as the reaction time of the hydrothermal treatment (second step) was the same for both composites, it hardly influenced particle growth [45]. 

The specific surface areas and the average pore diameter of the AC and the composites were determined by the nitrogen adsorption–desorption method and are presented in Table 1. The corresponding adsorption–desorption isotherms and pore size distribution curves are displayed in the Supplementary Material (Figures S1 and S2, respectively). Note that the shape of the obtained N_2_ isotherms agreed with those reported for porous carbon materials from a bio-based origin with the typical feature of an open hysteresis loop at the endpoint of the desorption curve. However, as reported in the literature, the reason for this phenomenon has not been clarified yet. Probably, the nitrogen molecules can cause deformation in the pore structure, producing an expansion of the pore volume during measurements at the test temperatures (−196 °C) [46]. 

The results shown in Table 1 reveal that the presence of α-FOD with a specific surface previously reported to be between 20 and 30 m^2^·g^−1^ [37] did not drastically affect the specific surface area of the AC. Several reports have shown that the addition of a photocatalyst with a relatively low surface area onto the AC surface can decrease the composite specific surface area value [8,24,47]. This can be attributed to the partial clogging of the active sites of the carbon by the photocatalyst particles, as observed in this work for composite CS6g, which is evidenced as well in the SEM images. Interestingly, for composite CS1g, the specific surface area is slightly higher than that of pure AC. This behavior has been reported by other authors [21,48] and can be explained by a slight modification of the meso/microtexture of the AC porous network during the treatment (acid hydrothermal process). For instance, AC derived from date stone and TiO_2_/BiOBr/Bi_2_S_3_/AC composites synthesized by Alikhani et al. [48] presented surface areas of 415 and 659 m^2^·g^−1^, respectively, which are comparable to the values reported in this study. Regarding the pore diameter, the results show a microporous structure for the AC, which is slightly increased by the addition of α-FOD, due to the partial blocking of the AC micropores [6].

### 2.2. Adsorption and Photocatalytic Tests

#### 2.2.1. Adsorption Isotherms

Phenol adsorption isotherms were constructed for the AC and the composites. The experimental results of the adsorption capacity at equilibrium (Q_ads_) vs. equilibrium concentration of phenol (C_eq_) were plotted and fitted to the nonlinear Langmuir (Equation (1)) and Freundlich (Equation (2)) models [49], which are the most commonly used expressions to describe the adsorption phenomenon.

(1) $$q_{e}=\frac{Q_{max}K_{L}C_{e}}{1+K_{L}C_{e}}$$

**Figure 3:**
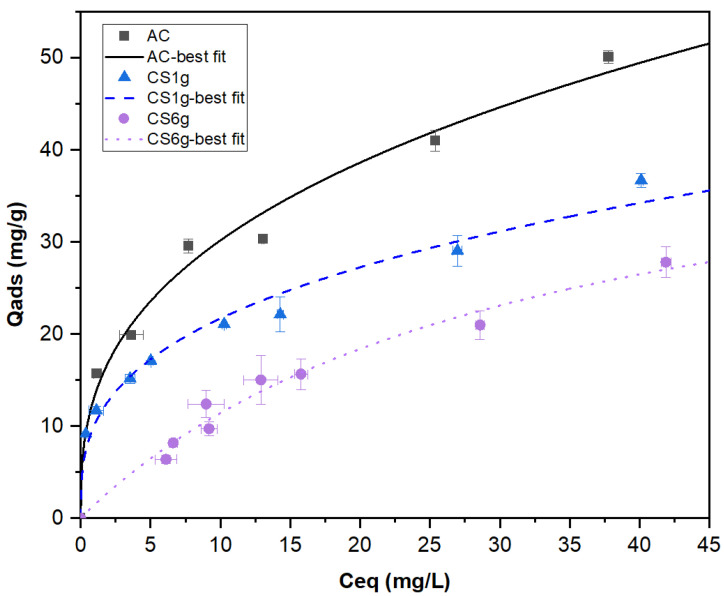
Adsorption isotherms for phenol adsorption by AC (■), composites obtained from the dissolution of 1 g (▲) and 6 g (⬤) of precursor in oxalic acid. The continuous, dashed, and dotted lines correspond to the best-fitted models.

(2) qe=KfCe1n

where q_e_ is the amount of phenol adsorbed per unit of mass of material, i.e., AC for pure adsorbent and composite for CS1g and Cs6g (mg·g^−1^), C_e_ is the equilibrium concentration (mg·L^−1^), Q_max_ is the maximum adsorption capacity (mg·g^−1^), K_L_ is the Langmuir constant (L·mg^−1^), and K_f_ and n are the Freundlich constants. The adsorption parameters of both models obtained for the three adsorbents are displayed in Table 2, and the experimental data as well as the best fit in each case are shown in Figure 3. The Freundlich model provided a better representation of the experimental points, which tended to indicate a multilayer coverage of phenol onto the adsorbents’ surfaces. The n > 1 values suggested that it was mainly a physical, therefore a reversible, adsorption phenomenon [50].

As expected, pure AC presented the highest adsorbent properties. Composites did not develop adsorption capacities directly proportional to the mass composition of AC. As an example, for an equilibrium concentration of 25 mg·L^−1^ pure AC adsorbed 41.8 mg·g^−1^ of phenol. A direct proportionality would lead to 38.5 mg·g^−1^ for CS1g and 24.2 mg·g^−1^ for CS6g, which are higher than those obtained experimentally, corresponding to 29.3 and 21.0 mg·g^−1^, respectively. While it did not strongly affect the specific surface area determined with N_2_ adsorption, the processing route, which probably limited and/or made access to the pore network more difficult, reduced slightly, if not significantly, the adsorption of larger molecules in solution. However, even if they were not directly proportional to the mass composition of AC, the composites still had interesting adsorption capacities. Adsorption functionality, the first objective, was preserved and remained effective.

#### 2.2.2. Photocatalytic Tests 

**Figure 4:**
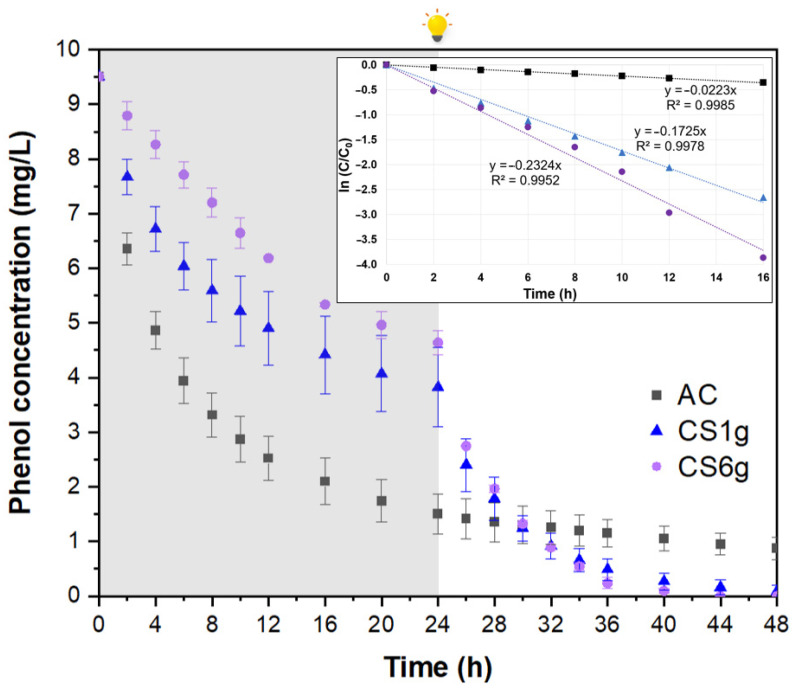
Phenol adsorption under dark conditions (first 24 h) and photocatalytic degradation under visible light (last 24 h) by using AC (■), and composites obtained from the dissolution of 1 g (▲) and 6 g (⬤) of precursor. Adsorbent dosage: 0.5 g/L. Inset: First-order kinetic equations of the photocatalytic degradation of phenol calculated with the first 16 h of visible irradiation.

Figure 4 displays the adsorptive and photocatalytic performance of the three adsorbents. It is well known that the absolute values of adsorbed and photodegraded quantities of a determined pollutant, as well as their corresponding rates, are, by their very nature, highly dependent on the operating conditions, such as the initial micropollutant concentration, solid particle concentration (AC or composites), irradiation intensity, stirring efficiency, etc. For this reason, it is important to observe that this analysis is performed merely for comparison purposes between the AC and the as-synthesized composites. In this sense, as expected from the adsorption isotherms, the AC was the best adsorbent. It removed the highest quantity of phenol from the liquid phase the fastest. The 84.2% of phenol was adsorbed by pure AC during the first 24 h, compared to around 59.8 and 51.2% for the composites CS1g and CS6g. In contrast, the phenol removal rate during the irradiated process decreased as follows: CS6g > CS1g > AC. Assuming, in a highly simplified way, that the rate of phenol degradation can be approximated by first-order kinetics, composite CS6g, the one with higher photocatalyst content, exhibited an apparent kinetic constant of 0.2324 h^−1^, which is 1.35-fold higher than the value for composite CS1g (0.1725 h^−1^). Also, it was confirmed that under irradiation, the AC has no photocatalytic properties and just continues to adsorb the phenol up to equilibrium (just for comparison, the kinetic coefficient calculated was 0.0223 h^−1^). It is important to note that the wide inferential error bars in Figure 4, corresponding to composite CS1g, are consistent with the heterogeneity of this material.

These outcomes prove that the two targeted composites’ functionalities were achieved. Phenol concentration profiles during the two different phases of the experiments (dark conditions and irradiation) definitively highlighted that the composites were able to adsorb and degrade phenol under visible light. This makes it possible to use them not only to adsorb the micropollutants, merely transferring them from one medium to another, but also to photodegrade them under visible or solar light into less toxic compounds.

The phenol removal capacity of various activated carbon-based composites with visible-light-driven photocatalytic activity has been reported in the literature (Table 3) in mg of phenol removed per gram of material. However, comparing the performance of the as-synthesized AC/α-FOD composites with other AC-based composites is extremely challenging because of differences between synthesized materials and testing conditions, such as the photocatalysts’ nature and particle size, the initial phenol concentration, irradiation sources and intensities, etc. For the most promising composite, CS6g, the calculated removal capacity after the first 24 h (adsorption under dark conditions) was about 9.7 ± 0.3 mg/g, which is lower than the value for pure AC (16.0 ± 0.6 mg/g). However, in the following 24 h (under visible light irradiation), the removal capacity of pure AC increased to 17.3 ± 0.3 mg/g, while CS6g reached 19.0 ± 0.1 mg/g, due to the photocatalytic effect. According to Table 3, overall, the phenol removal capacity of composite CS6g synthesized in this study and those described in the literature fall within the same range of order of magnitude, despite the low irradiation intensity used.

#### 2.2.3. Recycling Tests

**Figure 5:**
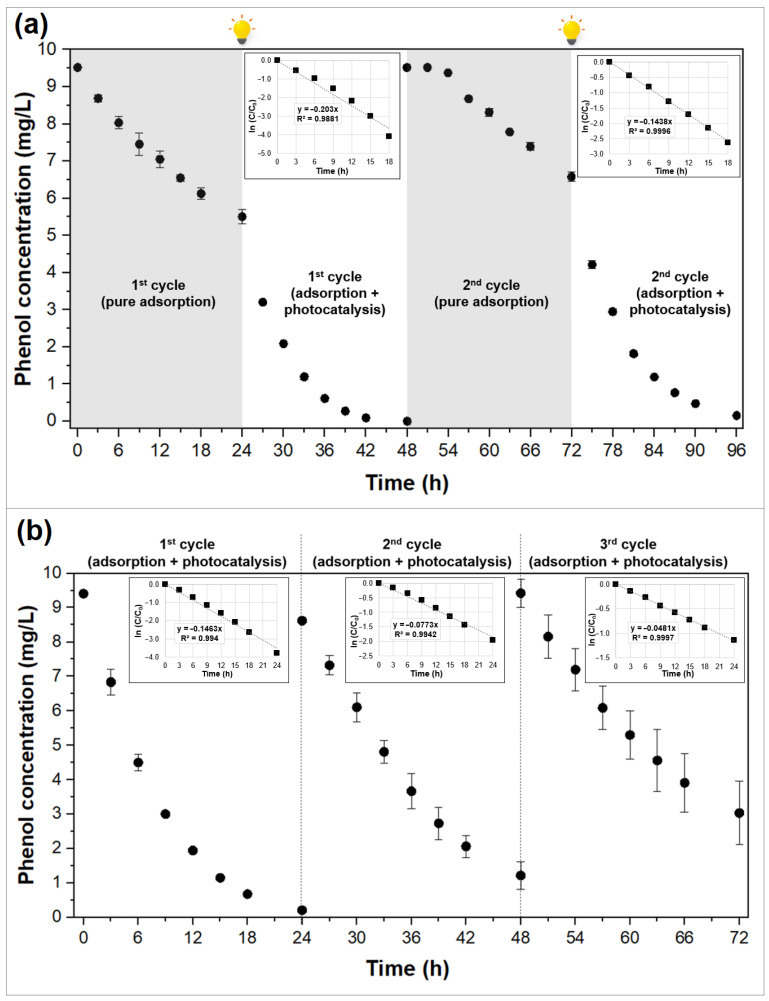
Regeneration tests for phenol removal by using composite CS6g. (**a**) Cycles of phenol removal under alternated cycles of 24 h under dark conditions and 24 h under visible light irradiation; inset: first-order kinetic equations. (**b**) Three consecutive 24 h cycles of photocatalytic degradation of phenol under visible irradiation; inset: first-order kinetic equations. Adsorbent dosage: 0.5 g/L.

Composite CS6g was selected for further recycling tests as it is the most homogeneous as-synthesized material. In addition, CS6g with the highest content of α-FOD has the best photocatalytic behavior, which is the most interesting property to evaluate in this work. The results of two types of recycling tests (described in Section 3) are shown in Figure 5. In Figure 5a, two 48 h cycles are displayed. During the first cycle, under dark conditions, around 42.2% of phenol was removed by adsorption, then under visible light irradiation, a combined phenomenon of adsorption–photocatalysis took place, and the phenol removal reached around 99.0%. During the second cycle, pure adsorption led to 30.9% of phenol removal, and after exposure to visible light, the removal percentage increased to 98.4%. These results reveal a loss in the adsorptive capacity of the composite; however, during the second cycle of adsorption–photocatalysis, the phenol concentration reached almost the same value as in the first cycle, near to 0, demonstrating its excellent photocatalytic capacity. The Raman spectra of the composite CS6g collected before and after the four cycles is displayed in Figure S3. Overall, the intensity of Raman bands diminishes, most likely as a result of surface leaching [51]; however, the Raman bands corresponding to α-FOD remain in their original positions, with values of 204.9, 245.9, 517.2, 584.1, 914.9, 1435.0, 1469.5, and 1706.7 cm^−1^ [52]. Therefore, these results confirm the stability of composite CS6g during phenol degradation. 

The results of the second regeneration experiment are displayed in Figure 5b. In this case, the three cycles correspond to tests under visible light irradiation, which means that adsorption and photocatalysis took place at the same time from the very beginning of the tests. As presented in Figure 5b, the removal efficiency of phenol declines from 97.7% in the first cycle to 85.8% in the second and 67.8% in the third cycle.

Whatever the operation mode of the cycling test, ideal cyclability is synonymous with identical concentration profiles. It is evident that in both recycling methods, the composite exhibits a decrease in the phenol removal efficiency. Nevertheless, all these results showed that the adsorption and photocatalytic treatment properties were partially maintained during successive cycles. Composites are reusable for a few cycles provided reduced performances are accepted. Asencios et al. [47], who also reported a loss in the degradation efficiency of phenol removal over the cycles when using a TiO_2_/AC composite, suggested additional treatments, such as the washing of the composite with pure water several times, to increase the composite regeneration. 

Whatever the cycling tests, phenol adsorption and phenol degradation by photocatalysis take place simultaneously and/or successively. Ideal cyclability is equivalent to the following: (i) total removal of the phenol adsorbed in the micropores when the photocatalytic treatment is activated and (ii) perfect composite durability. Among the very few studies dealing with AC/photocatalyst cyclabilities in the literature, the reduction in performance has been attributed to several causes: the loss of photocatalyst produced by the detachment of the α-FOD particles from the AC surface [21]; a decrease in the AC sites produced by the obstruction with α-FOD particles during the photocatalytic process [53]; the adsorption of the generated degradation intermediates on the active sites of the composite surface [24]; and/or the partial removal of the phenol adsorbed [54]. The answer is most likely a combination of these different interpretations. Additional experiments regarding the improvement of the attachment of the photocatalyst to the AC surface should be performed.

## 3. Materials and Methods

### 3.1. Materials 

Ferrotitaniferous black sand with an approximate composition of 0.6FeTiO_3_·0.4Fe_2_O_3_ [38] was used as the precursor for the composites’ synthesis. Oxalic acid dihydrate (99.0%) was purchased from DQI S.A. (Medellín, Colombia) and granular activated carbon (AC) from Wild Coast Organics Quito-Ecuador (obtained from coconut husks). Phenol (99.5%) from CDH was used for adsorption and photocatalytic tests. Ultrapure water from an Aquelix 5 system (18.2 MΩ-cm, Merck-Millipore, Burlington, MA, USA) was employed throughout all the experiments. 

### 3.2. Synthesis of the AC/α-FOD Composites

#### 3.2.1. Conditioning of the AC

The AC provided by the manufacturer had a particle size of ~4 mm, therefore, some conditioning was required before the composites’ synthesis. The AC was dried (80 °C for 8 h), milled (analytical mill, Cole-Parmer, Vernon Hills, IL, USA, 4301-00), and sieved (W. S. Tyler, Mentor, OH, USA), and the portion retained between sieves mesh #32 and #28 (corresponding to particle sizes between 500 and 595 µm) was used for the composites’ elaboration.

#### 3.2.2. Synthesis

**Figure 6:**
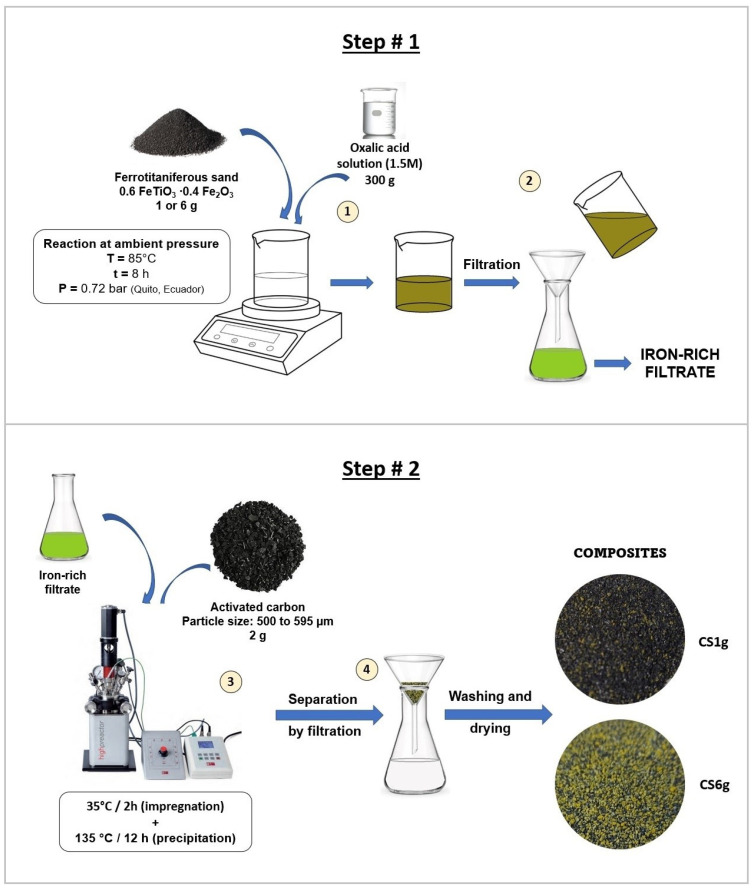
Schematic representation of the composites’ preparation.

The synthesis was performed in a two-steps process: first, the dissolution of the sand at ambient pressure, and second, the precipitation of α-FOD on the AC surface by using a green hydrothermal process, as shown in Figure 6. Briefly, either 1 g or 6 g of ferrotitaniferous sand was placed in a beaker with 300 g of a 1.5 M oxalic acid solution and magnetically stirred at 85 °C for 8 h. The product was separated by filtration and the filtrates were stored for their use in the second step. The resulting greenish filtrate corresponds to a ferric oxalate-rich solution, as reported in other studies [34,55]. The filtrate was heated up to 35 °C and placed in a high-pressure reactor system Berghof BR-500 (Baden-Württemberg, Germany) with stirring. Nitrogen gas (99.9% purity, Linde, Quito, Ecuador) was used for displacing the air present in the reactor vessel for 5 min before the hydrothermal synthesis. The stirring speed was set at 50 rpm, and reaction time and temperature were 2 h at 35 °C for the impregnation process, followed by 12 h at 135 °C to induce the reduction of the ferric oxalate to α-FOD [56], which precipitated onto the AC surface. After the hydrothermal reaction, and cooling of the system, the AC/α-FOD composites were washed until neutral pH, dried overnight at 80 °C, and sieved using the same meshes employed for the AC conditioning (mesh #32 and #28), ensuring composites with particle sizes between 500 and 595 µm. The composites were denoted as CS1g and CS6g for the composites obtained by dissolution of 1 g and 6 g of black sands, respectively. The dried composites were stored for further characterization and phenol removal tests. 

### 3.3. Characterization of the Materials

The composites CS1g and CS6g were characterized by XRPD to determine the phase composition of the impregnated photocatalyst by using a Bruker D2 Phaser X-ray diffractometer (Billerica, Massachusetts, USA) with CuK α radiation (λ = 1.54184 Å), using a 0.02° step size and 0.250 s/step, with a LYNXEYE XE-T detector (1D-mode) (Billerica, Massachusetts). Before the analysis, the composites were pulverized by mortar grinding. XRPD patterns obtained from the samples were compared to a standard X-ray diffraction card corresponding to monoclinic alpha ferrous oxalate dihydrate (α-FeC_2_O_4_·2H_2_O).

Scanning electron microscopy (SEM) images of the activated carbon and the two as-synthesized composites were obtained in an Aspex PSEM Express microscope (Delmont, Pennsylvania, USA) operated at 20 kV, while their specific surface areas were determined by the nitrogen adsorption–desorption method at low temperature and the BET equation, by using a Quantachrome Instruments Novatouch LX-1 (Boynton Beach, Florida, USA). The sample preparation for the adsorption–desorption measurements was as follows: the materials were degassed at 150 °C with 5 Torr vacuum for 3 h. 

The ratio of AC/α-FOD of the composites was determined by calcination of the samples and weighting of the final product. According to the literature, when ferrous oxalate is calcinated in an oxidative atmosphere it decomposes into hematite [56,57,58]. The test was performed in triplicate and consisted of calcinating the AC and AC/α-FOD composites samples in a furnace Carbolite 400 (Hope Valley, UK) for 2 h at 700 °C. The samples were weighed before and after the calcination process. Carbon samples were calcinated to determine the ash content. Assuming all the ferrous oxalate dihydrate has transformed into hematite, it was possible to estimate the approximate quantity of photocatalyst (α-FOD) that is initially present in the composite, by using the mass balance before and after calcination through Equations (3) and (4):

m_COM_ = m_AC_ + m_FOD_ (3)

m_CAL_ = m_ASH_ + m_HEM_ (4)

where m_COM_ is the composite mass, m_AC_ is the activated carbon mass, m_FOD_ is the ferrous oxalate dihydrate mass, m_CAL_ is the product mass after calcination, m_ASH_ is the ash mass, and m_HEM is_ the hematite mass. All the values of mass are expressed in grams.

### 3.4. Adsorption and Photocatalytic Tests

**Figure 7:**
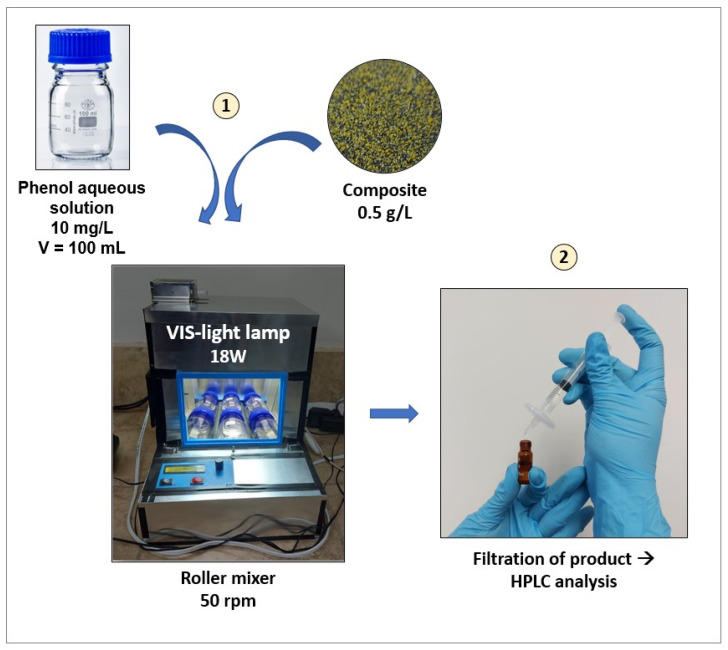
Set-up for the adsorption and photocatalysis tests.

Experiments for the adsorption isotherms were performed in duplicate using initial phenol concentrations ranging from 20 to 50 mg/L and adsorbent dosages from 0.2 to 2.0 g/L. The equipment employed was a homemade roller mixer with internal ventilation and an adjustable speed mechanism, which was set to 50 rpm. For all the experiments, 100 mL of phenol aqueous solutions were used. Figure 7 shows the setup for the phenol removal tests. Previous tests were performed, and 24 h was selected as a reasonable duration to approach equilibrium conditions. The isotherms were fitted to Langmuir and Freundlich nonlinear models and Excel solver software (version Office 365).

To study the adsorption and photocatalytic performance of the AC and the synthesized composites, a 48 h batch test was conducted. Thus, 100 mL of phenol solution with an initial concentration of 10 mg/L was placed into a transparent borosilicate bottle with 0.05 g of AC or composite. The experiment was conducted in the roller mixer, with the first 24 h of the experiment under dark conditions and the following 24 h under visible light exposition (Sylvania, LED panel SQ 18WDL, Shanghai, China) to show the adsorption and photocatalytic activity of the materials, respectively. 

Reuse tests were performed for the composite that showed the best photocatalytic performance. Two different ways of cycling were used; for all the experiments, 100 mL of a 10 mg/L phenol solution was placed in a transparent glass bottle with 0.05 g of composite. The first reuse experiment consisted of alternated cycles of 24 h under dark conditions followed by 24 h under visible light irradiation. After each irradiation cycle, the solution was decanted, and the supernatant was discarded; 100 mL of fresh 10 mg/L phenol solutions was added to the spent composite. Raman spectra of both fresh and used CS6g composite were obtained in a LabRAM HR Evolution Raman spectrometer equipped with a confocal microscope (HORIBA Scientific, Palaiseau, France) with a 633 nm laser irradiation set at 12.5 mW of power to confirm its stability. In the second reuse experiment, three cycles under irradiation were conducted. All the experiments were performed by duplicate; the average values are presented, and the standard deviations are shown in error bars. Phenol concentration was monitored periodically by High-Performance Liquid Chromatography (Agilent HPLC L1120 equipped with an Agilent Zorbax Eclipse Plus C18 column—4.6 × 150 mm, 5 µm particle size, Santa Clara, CA, USA).

## 4. Conclusions

In this work, two composites with different photocatalyst contents (8.3 and 42.7% *w*/*w*) were successfully produced through a simple two-step synthesis method by using ferrotitaniferous sands as a low-cost precursor. The two composites were characterized, and their adsorptive and photocatalytic properties under visible light irradiation were evaluated for phenol removal from synthetic solutions. The results revealed that monoclinic ferrous oxalate dihydrate (α-FOD) was obtained and attached effectively to the AC surface. The general trend is a better fit of the adsorption isotherms, with the Freundlich model revealing a multilayer adsorption phenomenon for heterogeneous surfaces. The photocatalytic tests showed that, as expected, the higher the photocatalyst content in the composite, the lower the adsorption efficiency, but the higher the rate of phenol photodegradation under visible light irradiation. Hence, for a 10 mg/L phenol solution, the adsorption was around 84.2% for AC, 59.8% for CS1g, and 51.2% for CS6g after 24 h under dark conditions. During the following 24 h, when the experiment was carried out under visible light irradiation, an additional 6.6% of phenol was removed by AC, whereas for composites CS1g and CS6g, the phenol removal reached 99.0 and 100.0%, with apparent kinetic coefficients of 0.1725 h^−1^ and 0.2324 h^−1^, respectively. The recycling experiments revealed a loss in the effectiveness of the composite through successive cycles. For this reason, future work on this material should focus on improving its recyclability. There is a question of elucidating the main reason for the loss of performance so that improvements can then be implemented. However, it was demonstrated that granular activated carbon obtained from coconut husk is not only an excellent adsorbent for phenol but also a suitable support for visible-light-active photocatalysts such as α-FOD. This novel composite with both adsorptive and photocatalytic properties can degrade as well as capture organic pollutants. Therefore, this promising material opens the possibility of being used in solar applications to address the intermittence of sunlight.

## Figures and Tables

**Table 1:** Surface characterization of the AC and the AC/α-FOD composites.

Adsorbent	Specific Surface Area (m^2^·g^−1^)	Average Pore Diameter (nm)
AC	314.3	1.71
CS1g	374.8	1.81
CS6g	251.8	2.48

**Table 2:** Adsorption isotherm parameters for the AC and the composites studied.

Adsorbent	Langmuir			Freundlich		
	Q_max_ (mg.g^−1^)	K_L_ (L·mg^−1^)	R^2^	K_F_ ((mg·g^−1^)·(L·mg^−1^)^1/n^)	n	R^2^
AC	52.28	0.17	0.957	13.29	2.81	0.988
CS1g	34.97	0.21	0.927	10.17	3.04	0.982
CS6g	47.24	0.03	0.989	2.69	1.60	0.977

**Table 3:** Photocatalytic test conditions and phenol removal efficiencies in this and other works.

Composite	Composite Dosage (g/L)	Phenol Initial Concentration (mg/L)	Particle Size (µm)	Removal Capacity and Irradiation Time	Irradiation Power (W)	Reference
AC/TiO_2_	0.5	50	NR	55 mg/g within 1 h (dark) + 4 h vis-irradiation	90	[47]
AC/Ag-AgCl	0.5	13	149	23 mg/g within 2 h (dark) + 2.5 h vis-irradiation	300	[24]
AC/fluorine doped TiO_2_	1	25	NR	15 mg/g within 30 min (dark + 8 h vis-irradiation)	250	[31]
AC/α-FOD (CS6g)	0.5	10	500–595	19 mg/g within 24 h (dark) + 24 h vis-irradiation	18	This work

NR: not reported.

## Data Availability

Dataset available on request from the authors.

## Supplementary Materials

The following supporting information can be downloaded at https://www.mdpi.com/article/10.3390/molecules29153690/s1, Figure S1. Nitrogen adsorption-desorption isotherms of the AC and AC/α-FOD composites; Figure S2. Pore size distribution curves of the AC and AC/α-FOD composites; Figure S3. Raman spectra of the as prepared and used composite CS6g after 4 cycles.
