# Supplementary material for: Adsorptive–Photocatalytic Composites of α-Ferrous Oxalate Supported on Activated Carbon for the Removal of Phenol under Visible Irradiation
Source: Molecules. 2024 Aug 4;29(15):3690. doi: 10.3390/molecules29153690 (PMC11314241; doi:10.3390/molecules29153690)
Supplement: Supplementary file 1 [file molecules-29-03690-s001.zip › molecules-3134615-supplementary.pdf]

Supplementary Material

Adsorptive-photocatalytic composites of α-ferrous oxalate supported on activated carbon for the removal of phenol under visible irradiation

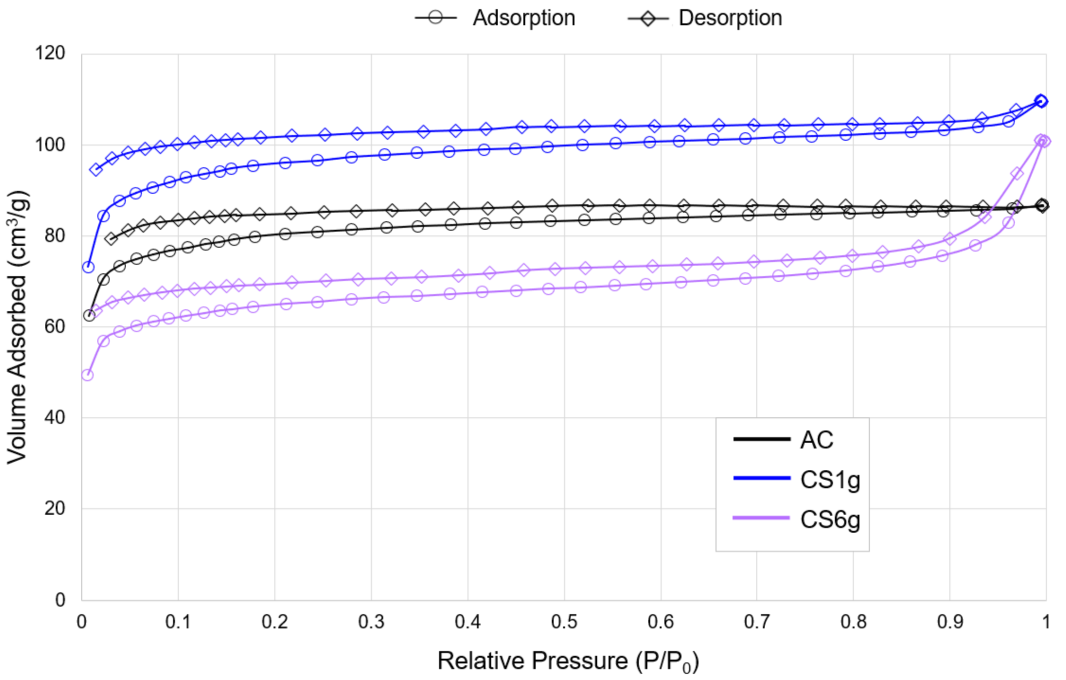

Figure S1. Nitrogen adsorption-desorption isotherms of the AC and AC/ α-FOD composites.

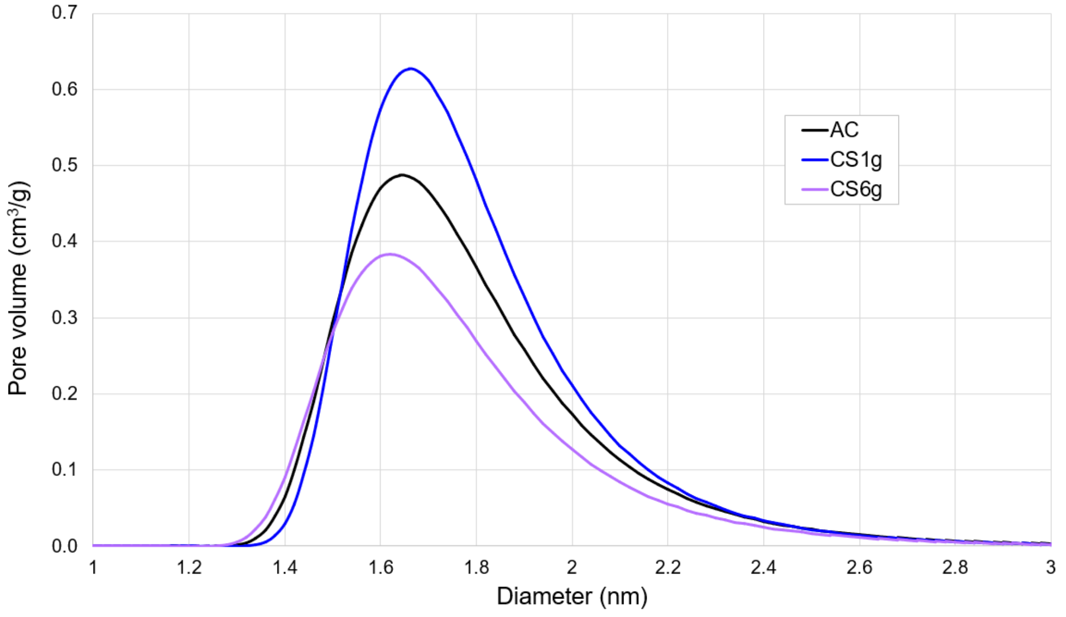

Figure S2. Pore size distribution curves of the AC and AC/ α-FOD composites.

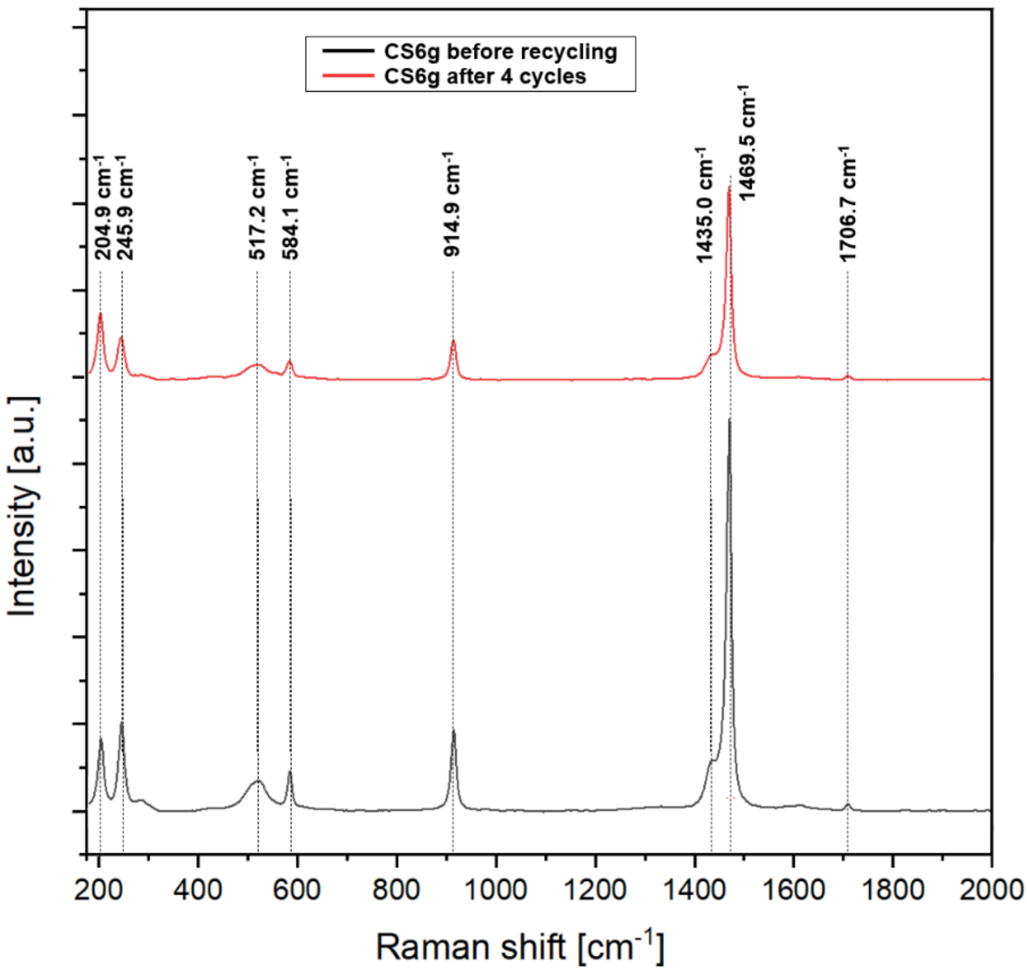

Figure S3. Raman spectra of the as prepared and used composite CS6g after 4 cycles.
